# Effect of Heme Oxygenase-1 on Melanoma Development in Mice—Role of Tumor-Infiltrating Immune Cells

**DOI:** 10.3390/antiox9121223

**Published:** 2020-12-03

**Authors:** Halina Was, Tomasz Cichon, Ryszard Smolarczyk, Bozena Lackowska, Agnieszka Mazur-Bialy, Magdalena Mazur, Agata Szade, Pawel Dominik, Milena Mazan, Jerzy Kotlinowski, Anna Zebzda, Anna Kusienicka, Claudine Kieda, Jozef Dulak, Alicja Jozkowicz

**Affiliations:** 1 Department of Medical Biotechnology, Faculty of Biochemistry, Biophysics and Biotechnology, Jagiellonian University, 30-387 Krakow, Poland; magda.filip@gmail.com (M.M.); agata.szade@uj.edu.pl (A.S.); dominik.pawel@gmail.com (P.D.); milena.mazan@ryvu.com (M.M.); j.kotlinowski@uj.edu.pl (J.K.); anna.kusienicka@doctoral.uj.edu.pl (A.K.); jozef.dulak@uj.edu.pl (J.D.); alicja.jozkowicz@uj.edu.pl (A.J.); 2 Laboratory of Molecular Oncology and Innovative Therapies, Military Institute of Medicine, 04-141 Warsaw, Poland; ckieda@wim.mil.pl; 3 Center for Translational Research and Molecular Biology of Cancer, Maria Skłodowska-Curie National Research Institute of Oncology, Gliwice Branch, 44-102 Gliwice, Poland; Tomasz.Cichon@io.gliwice.pl (T.C.); Ryszard.Smolarczyk@io.gliwice.pl (R.S.); 4 Department of Pathology, Oncology Center, 31-115 Krakow, Poland; z5niezab@cyf-kr.edu.pl; 5 Department of Ergonomics and Exercise Physiology, Faculty of Health Science, Jagiellonian University Medical College, 31-126 Krakow, Poland; agnieszka.mazur@uj.edu.pl; 6 Transplantation Centre, Jagiellonian University, 30-663 Krakow, Poland; a.zebzda@gmail.com

**Keywords:** cancer, melanoma, heme oxgenase-1 (HO-1), female, tumor-infiltrating immune cells

## Abstract

Objective: Heme oxygenase-1 (HO-1) is a cytoprotective, proangiogenic and anti-inflammatory enzyme that is often upregulated in tumors. Overexpression of HO-1 in melanoma cells leads to enhanced tumor growth, augmented angiogenesis and resistance to anticancer treatment. The effect of HO-1 in host cells on tumor development is, however, hardly known. Methods and results: To clarify the effect of HO-1 expression in host cells on melanoma progression, C57BL/6xFvB mice of different HO-1 genotypes, HO-1^+/+^, HO-1^+/−^, and HO-1^−/−^, were injected with the syngeneic wild-type murine melanoma B16(F10) cell line. Lack of HO-1 in host cells did not significantly influence the host survival. Nevertheless, in comparison to the wild-type counterparts, the HO-1^+/−^ and HO-1^−/−^ males formed bigger tumors, and more numerous lung nodules; in addition, more of them had liver and spleen micrometastases. Females of all genotypes developed at least 10 times smaller tumors than males. Of importance, the growth of primary and secondary tumors was completely blocked in HO-1^+/+^ females. This was related to the increased infiltration of leukocytes (mainly lymphocytes T) in primary tumors. Conclusions: Although HO-1 overexpression in melanoma cells can enhance tumor progression in mice, its presence in host cells, including immune cells, can reduce growth and metastasis of melanoma.

## 1. Introduction

Heme oxygenase-1 (HO-1, heat shock protein 32, encoded by *HMOX1*) is widely expressed by mammalian cells. HO-1 expression can be increased several-fold in response to various stimuli, including the HO-1 substrate heme, reactive oxygen species (ROS), nitric oxide species, prostaglandins, cytokines, heavy metals, and UV irradiation [1,2]. The main function of HO-1 is to catalyze heme degradation to biliverdin, free ferrous iron, and carbon monoxide. It is known that these by-products of HO-1 activity are important in the physiological response to oxidative stress [1]. In addition, HO-1 may accelerate angiogenesis through a variety of mechanisms, including induction of vascular endothelial growth factor (VEGF) transcription and increased production of pro-angiogenic carbon monoxide [3].

Overexpression of HO-1 has been reported in many cancers [4,5,6] and its levels can be further elevated in response to chemo-, radio- [7], or photodynamic therapy [8,9,10]. On the other hand, it was demonstrated that HO-1 might be involved in numerous drug-resistance types of various cancers [11,12,13,14]. Of importance, there are several lines of evidence showing that HO-1 may exert potent and comprehensive protumoral effects. In a previous study [15] we have shown that melanoma cells B16(F10) overexpressing HO-1 [B16(F10)-HO-1] displayed increased proliferation, stronger angiogenic potential, and were more resistant to oxidative stress induced by hydrogen peroxide. After intracutaneous inoculation, melanoma cells overexpressing HO-1 generated more packed tumors with augmented vascularization and higher production of VEGF. At the same time, mice injected with these cells showed reduced inflammatory edemas, decreased leukocyte infiltration and augmented levels of soluble receptor 1 of TNF-α (sTNF-α R1), whereas TNF-α itself was downregulated in tumor tissues. Altogether, HO-1 overexpression in melanoma cells resulted in shortened survival of hosts. In another model, B16(F10)-HO-1 cells injected intravenously formed more nodules in the lungs [15].

In contrast to this, data concerning the effect of HO-1 in host cells on tumor development are very limited. An equally important role of melanoma cells and the tumor microenvironment in tumor induction and growth has already been proposed for the development of melanoma and the escape of melanocytes from the control of keratinocytes [16]. The tumor stroma, which consists of many components, including the extracellular matrix, growth factors, cytokines, chemokines, blood vessels, inflammatory cells and fibroblasts, has a profound effect on tumor progression [17].

Moreover, melanoma is known to produce several factors that influence its environment, e.g., VEGF, basic fibroblast growth factor (bFGF), platelet-derived growth factor (PDGF), and transforming growth factors TGF-α and TGF-β [18].

HO-1 promoter polymorphisms described in humans result in different levels of HO-1 expression [19]. The absence of the short allele (i.e., more active promoter containing a lower number of glutathione thymidine repeats [(GT)n] GT repeats), which is correlated with higher HO-1 activity, significantly enhances the inflammatory reaction in the vessel wall and augments the risk for restenosis in patients undergoing balloon angioplasty [20] or stenting [21]. Of importance, the absence of the short allele also seems to correlate with a higher incidence of some tumors [22,23].

Therefore, the aim of our study was to investigate the effect of HO-1 expression in host cells on tumor growth. Our previous research showed that overexpression of HO-1 in melanoma cells accelerates tumor growth partially through the modulation of immune response [15]. Here, we focused mainly on tumor-infiltrating immune cells. We used C57Bl/6xFvB mice of different HO-1 genotypes, HO-1^+/+^, HO-1^+/−^ and HO-1^−/−^, injected with B16(F10) melanoma cells. After intracutaneous and intravenous injection, we assessed effects of HO-1 expression levels in host cells on kinetics of tumor growth, metastasis, and tumor-related inflammation.

## 2. Materials and Methods

### 2.1. Reagents

Triton X-100, phenylmethyl sulfonyl fluoride, leupeptin, aprotinin, and Bicinchoninic Acid Assay kit were purchased from Sigma (Poznan, Poland). Acetone was procured from Polskie Odczynniki Chemiczne. Isoflurane was obtained from Abbott Laboratoria (Warszawa, Poland). Enzyme-Linked Immunosorbent Assay (ELISA) kits for mouse VEGF—Vascular Endothelial Growth Factor, and KC—keratinocyte chemoattractant chemokines CXCL1/2, mouse homologues of human growth-regulated oncogenes (GRO), were procured from R&D Systems (Minneapolis, MN, USA). Goat serum was purchased from GE Healthcare (Buckinghamshire, UK). Mouse monoclonal anti-proliferating cell nuclear antigen (PCNA) antibody (clone PC10) was obtained from Dako (Glostrup, Denmark). Goat anti-mouse-immunoglobulin conjugated with Alexa Fluor 546 was obtained from Invitrogen (Eugene, OR, USA). 4′,6-diamidino-2-phenylindole (DAPI) medium was procured from Victor Laboratories (Peterborough, UK). A Fluorescein FragEL™ DNA Fragmentation Detection kit (terminal deoxynucleotidyl transferase dUTP nick end labeling, TUNEL) was obtained from Calbiochem (Darmstadt, Germany). Antibodies against CD3, CD11b, CD19, CD45, Gr-1, and NK 1.1, the BD™ Cytometric Bead Array (CBA) Mouse Inflammation kit and 2,2′,2″,2‴-(Ethane-1,2-diyldinitrilo)tetraacetic acid (EDTA) were purchased from BD Biosciences (San Jose, CA, USA). The Luciferase Assay System was obtained from Promega (Madison, WI, USA).

### 2.2. Cell Culture

B16(F10) murine melanoma cell lines were purchased from American Type Culture Collection (ATCC). Cells were maintained in RPMI 1640 medium containing 10% fetal bovine serum (FBS), glutamax (2 mmol/L), penicillin (100 U/mL), streptomycin (0.1 μg/mL) and ciprofloxacin (10 μg/mL) at 37 °C, in a humidified atmosphere with 5% CO_2_. PT67 packaging cell line, used for the production of retroviral vectors, were cultured in DMEM High Glucose medium with 10% FBS, glutamax (2 mmol/L), penicillin (100 U/mL), streptomycin (0.1 μg/mL) and ciprofloxacin (10 μg/mL).

### 2.3. Preparation of Plasmids

Plasmid M13 was a kind gift from Dr. Christine Brostian (Medical University of Vienna, Vienna, Austria) and pBMN-EGFP-I-Luc was a gift from Dr. Magnus Essand (Uppsala University, Uppsala, Sweden). Plasmids were isolated from bacteria culture using an QIAfilter Plasmid isolation kit. The integrity of DNA was determined by electrophoresis in 1% agarose gel.

### 2.4. Preparation of Retroviruses

Retroviral vectors were produced in PT67 cells, which were cultured on the 6 cm in diameter dishes. The transfection mixture contained 3 μg of pBMN-EGFP-I-Luc, 1.5 μg of helper plasmid M13, 30 μL of SuperFect^®^ and filled up to 150 µL with DMEM HG empty medium. The mixture was then incubated for 10 min at room temperature. In the next step, 1 mL of complete medium was added. Then, PT67 cells, which had been washed twice with phosphate buffered saline (PBS), were exposed to the transfection mixture. After 5 h of incubation, the culture medium was replaced with a new one. On the next day, the medium was changed for 2 mL of fresh medium with HEPES. Two days after transfection, 0.5 mL of fresh RPMI 1640 complete medium together with 0.5 mL of the medium from transfected PT67 cells was added to the B16(F10) cells. After 3 days, the transfection efficacy was less than 5%, as checked under the fluorescence microscope according to presence of EGFP protein. Several passages of EGFP-positive colonies and double cell sorting using the MoFlo^®^ flow cytometer (Dako Cytomation, Glostrup, Denmark) allowed us to establish the B16(F10)(EGFP-I-Luc) cell line with the purity level of 99%. Selected cells were also checked for luciferase activity.

### 2.5. Animals

Eight–ten-week-old HO-1^−/−^, HO-1^+/−^ and HO-1^+/+^ males and females (C57BL/6 × FvB background) were used. They were generated from breeding pairs of HO-1^+/−^ mice transferred to Krakow from the original colony maintained at Birmingham (a kind gift of Dr. Anupam Agarwal, University of Alabama). Experiments were carried out in a conventional animal house, with veterinary care and asymptomatic animals. Animals were maintained in conventional cages (up to 5 animals) under controlled environmental conditions (10L:14D photoperiod, temperature: 22 ± 2 °C, humidity 45–65%), with a wood cutting bed, under a minimum of extraneous disturbance. Cages had enriched the environment with teethers and nesting material.

Studies were done in randomized and blinded manner. Mice were placed in cages not by genotypes, but according to litters. They were ear tagged and assigned to numbers. Measurement of tumor size or bioluminescence was assigned to the number. The decoding of the numbers took place after the measurement. Each of three experimental groups consisted of 4–10 mice. All mouse experiments were approved by the Institutional Animal Care and Use Committee at the Jagiellonian University with permit number 02/2007.

In the first set of experiments, aimed at analyzing the growth of primary tumors and mortality, mice were injected with 100 μL of saline containing 2 × 10^5^ B16(F10) cells intracutaneously. Using a caliper, the tumor diameters were measured every day, and tumor volumes were determined using the following formula: *V* = *D* × *d*^2^ × 0.52 (*V*, tumor volume; *D*, the biggest dimension; *d*, the smallest dimension). At the end of the experiments (different time points), mice were euthanized by ketamine/xylazine injection. All animal treatments were carried out in the treatment room, not in breeding/experimental room.

In the second set of experiments, aimed at analyzing the growth of secondary tumors, mice were inoculated with B16(F10)(EGFP-I-Luc) cells into the tail vein (2 × 10^5^ cells in 100 μL of saline). On days 7, 14 and 21, the bioluminescence signals were monitored using the IVIS system Lumina (Caliper Life Science, Hopkinton, MA, USA). Luciferase activity in mice anesthetized with isoflurane were determined by i.p. injection of 100 μL of 15 mg/mL d-luciferin followed by a 5-min exposure IVIS imaging. IVIS^®^ Living Image software was used to grid the imaging data and integrate the total bioluminescence signals in each boxed region. At 21 days after tumor cells’ injection, mice were euthanized by ketamine/xylazine injection. All animal treatments were carried out in the treatment room, not in breeding/experimental room.

To apply the 3 R rule in our in vivo experiments, we took the following steps: 1. the studies presented here are continuation of our previous in vitro and in vivo tests [15,24] (replacement), 2. for measuring primary tumors, we used a caliper, and for secondary tumors, measuring IVIS bioluminescence (reduction and refinement), 3. to avoid the non-specific effects of pharmacological inhibitors HO-1, we used genetically modified mice of different genotype (reduction and improvement), 4. we took care of the location of animals and reduced their stress during treatments (refinement).

### 2.6. Immunohistochemistry of Proliferating Cell Nuclear Antigen (PCNA)

Frozen 6-μm tumor sections were fixed in chilled acetone (4 °C) for 10 min and dried at room temperature. Non-specific binding was blocked with 10% goat serum in Tris-buffered saline (TBS) (1 h at room temperature). Then, tumor sections were incubated at 4 °C overnight in a humid chamber with mouse monoclonal anti-PCNA antibody diluted 1:200 in TBS. After washing away unbound antibodies, slides were incubated with goat anti-mouse immunoglobulin labeled with Alexa Fluor 546, diluted 1:200 in TBS. PCNA-positive cells were identified by red dots inside the nucleus. The nuclei were stained with DAPI dye (330–380 nm). To demonstrate the specificity of the staining, negative controls were prepared, where the sections were incubated without the primary antibody. At least 500 cells per region and 10–16 random regions were analyzed.

### 2.7. TdT-Mediated Fluorescein FragEL™ DNA Fragmentation Detection (TUNEL)

Frozen 6-µm sections of melanomas were stained using the FragEL™ fluorescein DNA fragmentation detection kit. Sections were incubated with Proteinase K for 10 min at room temperature and with final deoxynucleotidyltransferase (TdT) equilibrium buffer for 30 min at room temperature. It was followed by incubation with labeled TdT reaction mixture at 37 °C for 1.5 h in a humidified chamber. The total cell population was visualized using a DAPI filter (330–380 nm). Fluorescein-labeled nuclei were analyzed using a standard 465–495 nm fluorescein filter. To demonstrate the specificity of the staining, tumor sections were incubated without the labeled TdT reaction mixture. At least 500 cells per region and 10–16 random regions were analyzed.

### 2.8. Flow Cytometry of Tumor-Infiltrating Leukocytes

To prepare single-cell suspensions from the solid tumors, the tumor tissue was cut into small pieces with a pair of scissors. Then, the cell suspension was filtered and washed twice. Cells (~10^6^) were resuspended in 100 μL of buffer (PBS, 2% FBS) and incubated with 1 μg of appropriate FITC-, PE-, and PerCP-labeled mAbs for 30 min at 4 °C. After incubation, cells were washed twice and resuspended in 0.5 mL of the buffer. BD FACSCalibur flow cytometer and BD CellQUEST software (BD Biosciences) were used for data acquisition. Anti-GR-1, and anti-CD11b antibodies were used in a three-color combination with anti-CD45 to identify granulocytes and macrophages. Anti-CD3, anti-CD19, and anti-NK 1.1 antibodies were used in a two-color combination with anti-CD45 to identify different subpopulations of lymphocytes. Each analysis included 10,000 events. The results are shown as a percentage of the total number of cells of all identified populations.

### 2.9. Measurement of Protein Level

Total protein concentrations were determined using a bicinchoninic acid protein assay kit, according to the manufacturer’s instructions.

### 2.10. Measurement of Proinflammatory and Proangiogenic Protein Levels

Concentrations of inflammatory proteins: IL-6, IL-10, IL-12p70, TNF, INFγ and MCP-1 in blood serum were measured using the cytometric BD™ CBA Mouse Inflammation kit according to the vendor’s instructions. BD FACSCalibur flow cytometer, BD CellQUEST and BD CBA software (BD Biosciences) were used for data acquisition. Levels of proangiogenic and immunomodulatory factor VEGF and KC were measured by colorimetric sandwich ELISA according to the manufacturer’s protocols. Results were normalized to total protein concentration.

### 2.11. Analysis of Hematological Parameters

Samples of blood were harvested from murine tails three weeks after intravenous injection with B16(F10)(EGFP-I-Luc) melanoma cells. Healthy animals of the same age were used as controls. A total of 20 μL of blood was transferred to EDTA-coated tubes and analyzed immediately for: WBC (white blood cells), RBC (red blood cells), HGB (hemoglobin), HCT (hematocrit), PLT (platelets), MCV (mean corpuscular volume), MCH (mean corpuscular hemoglobin), and MCHC (mean corpuscular hemoglobin concentration) using an ABC Vet device (HORIBA ABX, Warszawa, Poland).

### 2.12. Measurement of Luciferase Activity in Tissue Homogenates

Tissues specimens (~0.5 cm^3^) were homogenized using TissueLyzer machine (Qiagen, Germany) in 200 μL of Lysis Reporter Buffer. After centrifugation (15 min, 20,000× *g*, 4 °C), 20 μL of harvested lysates were mixed with 50 μL of substrate and levels of chemiluminescence were measured using Microlumat LB96P machine and *WinGlow* software (EG&G Berthold, Bad Wildbad, Germany). The results were normalized to total protein concentration.

### 2.13. Statistics

All in vitro experiments were performed in duplicates, triplicates or tetraplicates and repeated independently at least twice as indicated. All results are presented as mean ± standard error (SE). Most statistical analyses were carried out with the t-Student test (for comparison of two independent groups) or one-way ANOVA with a posteriori Tukey test (for comparison of more groups). In case of non-normal distribution, the Mann–Whitney test (for comparison of two groups) or nonparametric analysis of Kruskal–Wallis variance with Dunn’s test (for comparison of more groups) were used. In the studies of tumor growth kinetics, Student’s t-test for pairs was used. Proportions were analyzed using the chi^2^ test (for *N* > 10) or Fisher’s exact test (for *N* < 10). Survival analyses were performed using the Kaplan–Meyer test. When comparing results, differences for *p* < 0.05 were considered statistically significant, while *p* < 0.2 was considered to show a statistically significant tendency/trend. The following labels of statistical significance were used for figures: * *p* < 0.05; ** *p* < 0.01; *** *p* < 0.001.

## 3. Results

### 3.1. Lower Level of HO-1 Expression in Mice Accelerates the Growth of Primary Tumors

**Figure 1:**
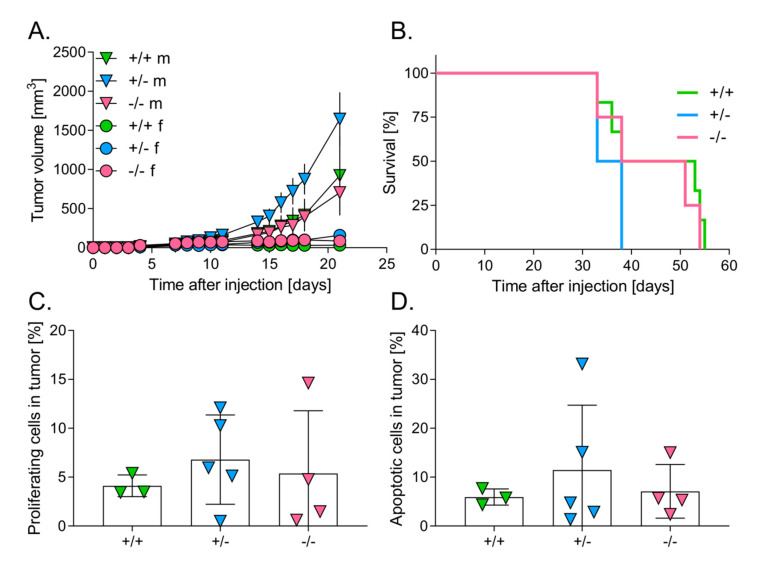
Effect of Heme oxygenase-1 (HO-1) expression in mice on survival and primary tumor growth. (**A**) Tumor volume in males (m) (*N* = 4–10) and females (f) (*N* = 4–10) of different HO-1 genotype: +/+, +/− or −/−. (**B**) Survival of males (*N* = 4–6). (**C**) Percentage of proliferating cells (proliferating cell nuclear antigen (PCNA)/DAPI-positive cells). (**D**) Percentage of apoptotic cells (TUNEL/DAPI-positive cells). Each point represents mean ± SE (*N* = 3–5).

To investigate the effect of HO-1 expression in host cells on growth of primary tumors, C57BL/6 x FvB males and females of different HO-1 genotypes (HO-1^+/+^, HO-1^+/−^, HO-1^−/−^) were injected intracutaneously with B16(F10) melanoma cells (2 × 10^5^ cells/mouse). Interestingly, in some animals, after the initial growth, we observed a regression of tumors. It was rather not due to the HO-1 genotype (28.57% in HO-1^+/+^, 17.65% in HO-1^+/−^ and 25% in HO-1^−/−^, *p* > 0.5), but to sex (33.3% in females vs. 11.1% in males, *p* = 0.13) *(*Figure 1A, Figure S1A,B).

Subsequently, the analysis of tumor growth revealed strong and statistically significant differences between females and males of all genotypes starting from the 10th day (*p* < 0.05) (Figure 1A, Figure S1A,B). Females grew very small tumors. In particular, starting from the 7th day after injection, tumor growth was completely inhibited in the wild-type females *(*Figure 1A, Figure S1B*),* whereas HO-1^+/−^ (Figure 1A, Figure S1B) and HO-1^−/−^ females (Figure 1A, Figure S1B) generated much smaller tumors in comparison to the corresponding males. There were no statistically significant differences in tumor growth between males of different HO-1 genotypes, although heterozygous animals (HO-1^+/−^) seemed to produce the biggest tumors (*p* = 0.243) (Figure 1A, Figure S1B). Kinetics of tumor growth were, however, similar in wild-type (HO-1^+/+^) and HO-1 (HO-1^−/−^)-deficient males (Figure 1A, Figure S1A).

### 3.2. Level of HO-1 Expression in Mice Does Not Influence the Survival of Mice

C57BL/6 x FvB males of different HO-1 genotypes were injected intracutaneously with B16(F10) melanoma cells (2 × 10^5^ cells/mouse). There were no statistically significant differences in survival between the groups (Figure 1B, *p* = 0.17). However, the value of median was the lowest in case of heterozygous (35.5 days) comparing to wild-type (45.5 days) and HO-1-deficient animals (44.5 days) (Figure 1B), which seemed to correspond to tumor growth (Figure 1A).

Next, we assessed the number of proliferating and apoptotic cells within the tumors. To elucidate the number of proliferating cells in tumors, frozen specimens were prepared, and then immunofluorescently stained for PCNA antigen. Cell nuclei were stained using DAPI dye. There were no significant differences in proliferating cells between HO-1 genotypes (Figure 1C). Frozen specimens were used also to evaluate the number of apoptotic cells in tumors, with a Fluorescein FragEL™ DNA Fragmentation Detection kit. There were no significant differences in apoptotic cells between genotypes either (Figure 1D).

### 3.3. Melanomas Growing in Mice with Higher HO-1 Expression Were More Heavily Infiltrated by Leukocytes

**Figure 2:**
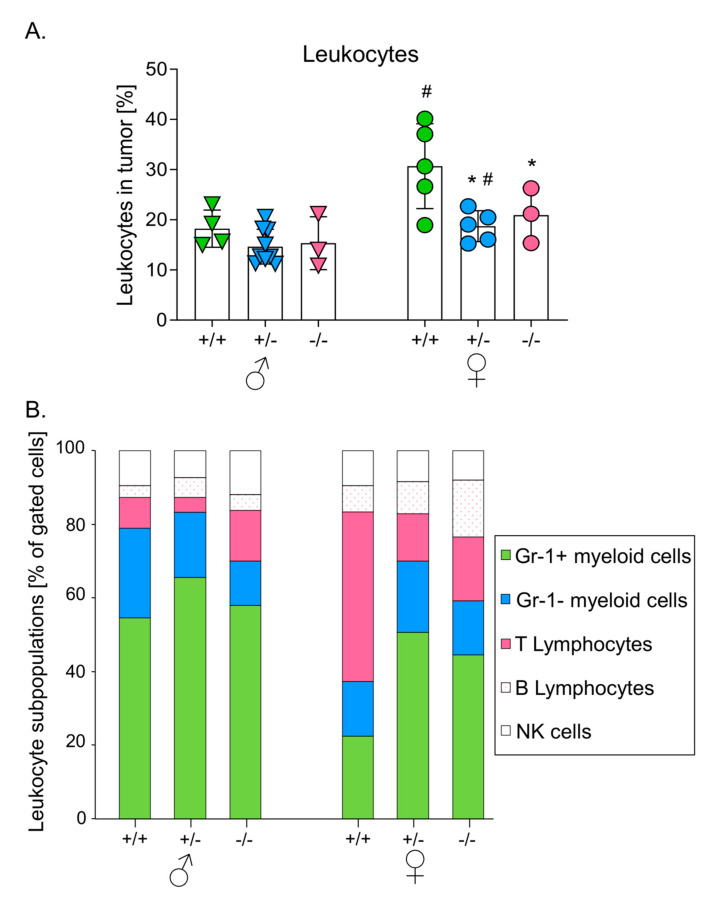
Effect of HO-1 expression in mice on leukocytes infiltration in primary tumors. (**A**) Percentage of leukocytes (CD45^+^ cells) in tumors. Cytometric analysis. Each point represents mean ± SE (*N* = 3–9). * *p* < 0.05 vs. HO-1^+/+^ mice, # *p* < 0.05 ♂ vs. ♀. (**B**) Subpopulations of infiltrating leukocytes: CD11b^+^Gr1^+^ myeloid cells, CD11b^+^Gr1^−^ myeloid cells, CD3^+^ T lymphocytes; CD19^+^ B lymphocytes; Nk 1.1^+^ NK cells. Cytometric analysis.

To define the influence of host genotype on the response of immune system to growing melanoma, analysis of leukocyte infiltration was performed using flow cytometry. The highest proportion of CD45 positive cells was found in tumors in wild-type animals (25.15 ± 3.24%) in comparison to HO-1^+/−^ (16.10 ± 1.05, *p* < 0.05) and HO-1^−/−^ (18.14 ± 2.55, *p* = 0.09) counterparts. In correspondence to the inhibition of tumor growth in HO-1^+/+^ females, the highest proportion of leukocytes was found in tumors in these animals (30.69 ± 4.22%, *p* < 0.05 in comparison to HO-1^+/−^, HO-1^−/−^ females and to males of all genotypes) (Figure 2A). Tumors in heterozygous females were also more heavily infiltrated by leukocytes (18.71 ± 1.54%) than in corresponding males (14.66 ± 1.23%, *p* < 0.05). Only in case of HO-1^−/−^ mice were there no statistically significant differences in tumor infiltration between females and males (*p* = 0.136) (Figure 2A).

Further analysis showed that subpopulations of infiltrating leukocytes differed between genotypes. Generally, the myeloid cells (including CD11b^+^Gr1^+^ and CD11b^+^Gr1^−^ cells) dominated among leukocytes infiltrating the growing tumors, with CD11b^+^Gr1^+^ cells representing more than 40% of the identified cells (Figure 2B). The only exception were wild-type females, where the predominant cells were T cells (67.37 ± 9.21% of gated cells), whereas CD11b^+^Gr1^+^ cells accounted for only 22.42 ± 3.34% of gated cells (Figure 2B).

### 3.4. HO-1 Expression in Mice Influences Levels of Serum Cytokines after the Intracutaneous Inoculation of Melanoma

To investigate the effect of HO-1 on tumor-related inflammation, we measured the concentration of IL-6, IL-10, IL-12, MCP-1 and IFNγ in serum using the BD™ CBA Mouse Inflammation kit. KC and VEGF were measured using ELISA.

**Figure 3:**
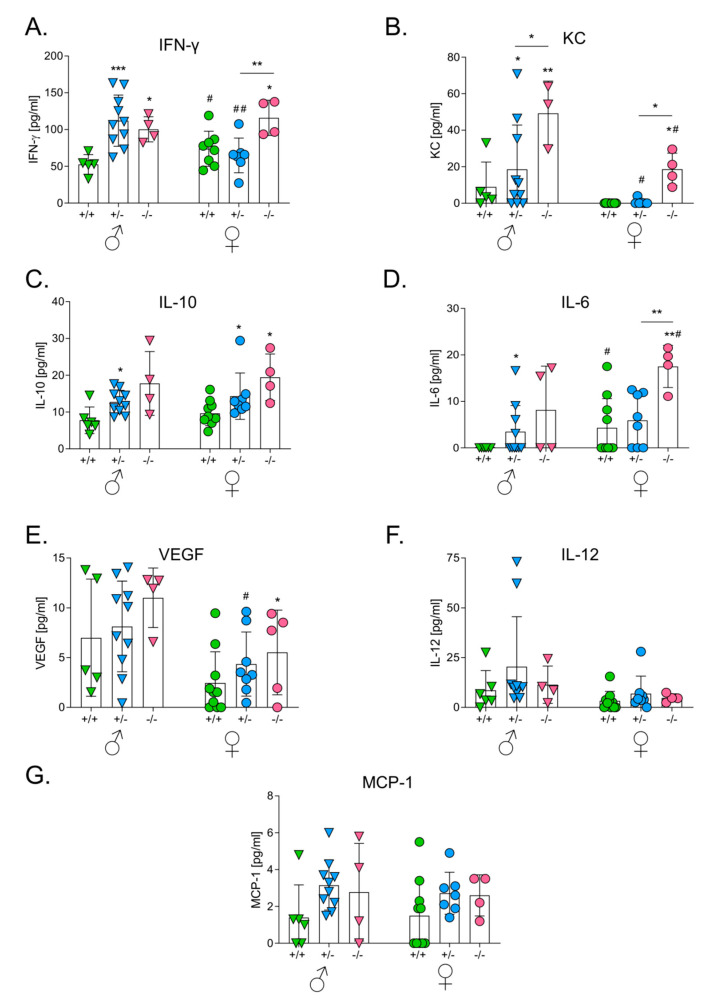
Effect of HO-1 expression in mice on concentrations of proangiogenic and proinflammatory cytokines in sera after intracutaneous injection with melanoma cells. (**A**) Concentration of IFN-γ. Cytometric analysis. (**B**) Concentration of keratinocyte chemoattractant (KC). ELISA. (**C**) Concentration of IL-10. Cytometric analysis. (**D**) Concentration of IL-6. Cytometric analysis. (**E**) Concentration of vascular endothelial growth factor (VEGF). Cytometric analysis. (**F**) Concentration of IL-12. Cytometric analysis. (**G**) Concentration of MCP-1. ELISA. Each bar represents mean ± SE (*N* = 4–10; * *p* < 0.05, ** *p* < 0.01, *** *p* < 0.001 vs. HO-1^+/+^ mice, # *p* < 0.05, ## *p* < 0.01♂ vs. ♀).

The effect of the HO-1 genotype was visible in the case of IFNγ (Figure 3A), KC (Figure 3B) and IL-10 (Figure 3C), both in males (*p* < 0.01 for IFNγ, *p* < 0.05 for KC, *p* < 0.05 for IL-10) and females (*p* < 0.01 for IFNγ, *p* < 0.001 for KC, *p* < 0.05 for IL-10). Concentrations of these cytokines were significantly elevated in HO-1^−/−^ mice. A similar tendency was observed for IL-6 (Figure 3D) and VEGF (Figure 3E) (*p* < 0.01 only in females). There was no significant influence of HO-1 on concentrations of IL-12 (Figure 3F) and MCP-1 (Figure 3G).

Sex was not an independent factor affecting the production of proinflammatory cytokines although there were some differences between females and males in the case of KC (Figure 3B), VEGF (Figure 3E) and IL-6 (Figure 3D).

### 3.5. HO-1 Expression in Mice Inhibits Tumor Growth in Lungs

**Figure 4:**
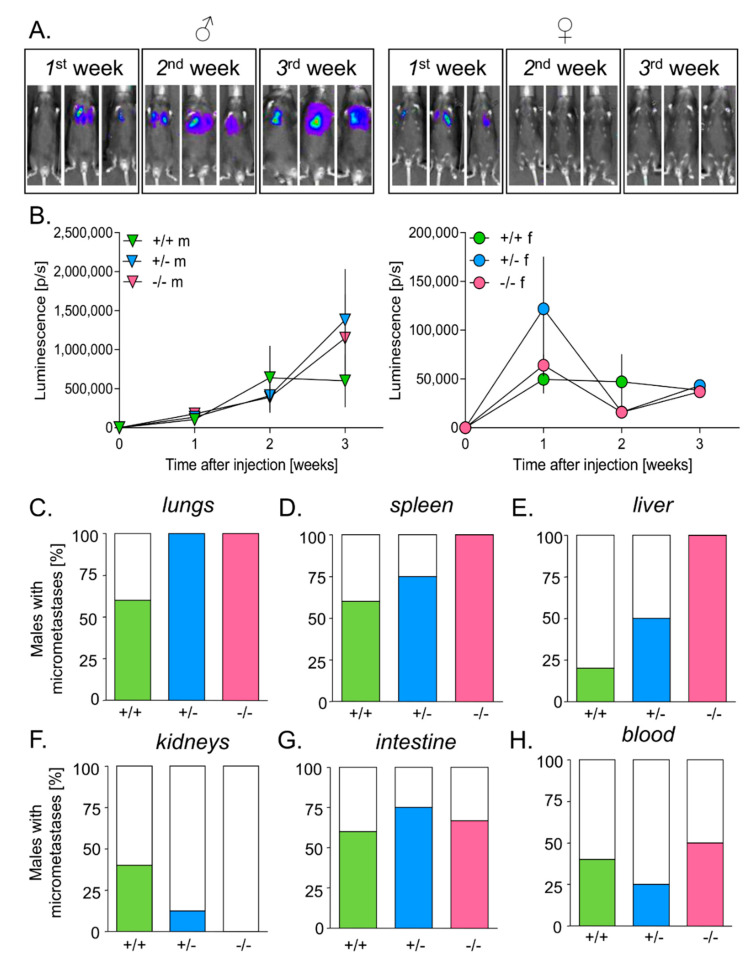
Effect of HO-1 expression in mice on tumor growth in lungs and the presence of micrometastases. (**A**) Tumor growth in males (♂) and females (♀) after 1, 2 and 3 weeks. IVIS measurements. Representative pictures. (**B**) Tumor growth in males (m) (*N* = 5–8) and females (f) (*N* = 6–7) of different HO-1 genotypes: +/+, +/− or −/− (signal from females is much weaker—please note the scale). Each bar represents mean ± SE. Percentage of males having micrometastases in: (**C**) lungs, (**D**) spleen, (**E**) liver, (**F**) kidneys, (**G**) intestine, and (**H**) blood. Measurements of luciferase activity in tissues’ homogenates.

To investigate the effect of HO-1 expression in host cells on the homing of melanoma cells to the lungs, B16(F10)(EGFP-I-Luc) cells were injected intravenously (2 × 10^5^ cells/mouse) to C57BL/6 x FvB males and females of different HO-1 genotypes. Activity of luciferase in the injected melanoma cells was analyzed by IVIS^®^ Lumina to monitor tumor growth in living animals (Figure 4A).

Like in the case of intradermal inoculation, we found significant differences between males and females. Measurements on the 1st week post injection showed that melanoma cells efficiently settled in the lungs of all females. Subsequently, the tumor growth was inhibited completely (Figure 4A,B) and was similar in all females, regardless of the genotype. Males grew significantly bigger tumors than females (Figure 4A,B). Interestingly, HO-1^+/+^ males seemed to inhibit tumor growth starting from the 14th day, whereas tumors in HO-1^+/−^ and HO-1^−/−^ males continued to grow onward (Figure 4B).

Additionally, luciferase activity was measured in lysates prepared from blood cells and fragments of lungs, spleen, liver, intestine and kidneys to detect circulating cancer cells and micrometastases. In accordance with the measurements carried out in animals in vivo, no signal was detected in lysates prepared from females’ lungs. In contrast, 60% of HO-1^+/+^ males and all HO-1^+/−^ and HO-1^−/−^ males developed lung micrometastases (Figure 4C) (*p* = 0.118). Moreover, females did not develop micrometastases in other organs, whereas males did (*p* < 0.001).

As opposed to HO1^+/+^ and HO-1^+/−^ males, all HO-1^−/−^ males displayed micrometastases in the spleen (Figure 4D) and liver (Figure 4E) but not in the kidneys (Figure 4F). No effect of HO-1 expression on the appearance of micrometastases in the intestine (Figure 4G, *p* > 0.5) or on circulating melanoma cells in the peripheral blood (Figure 4H, *p* > 0.5) was noticed.

### 3.6. HO-1 Expression in Mice Influences Levels of Serum Cytokines after the Intravenous Inoculation of Melanoma

**Figure 5:**
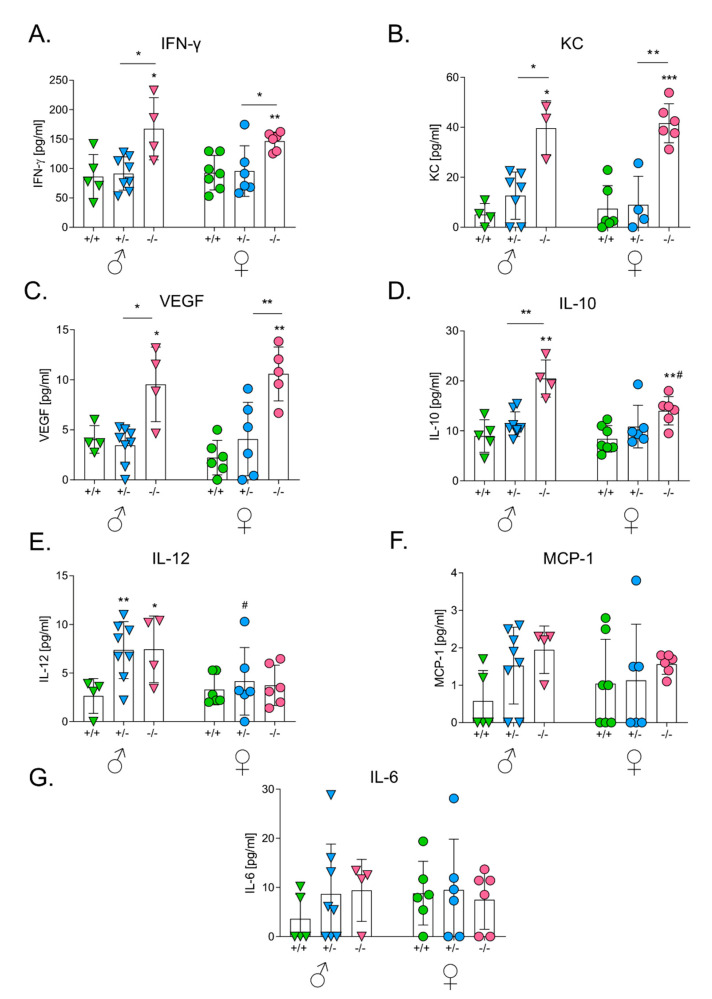
Effect of HO-1 expression in mice on concentrations of proangiogenic and proinflammatory cytokines in sera after intravenous injection with melanoma cells. (**A**) Concentration of IFN-γ. Cytometric analysis. (**B**) Concentration of KC. ELISA. (**C**) Concentration of VEGF. ELISA. (**D**) Concentration of IL-10. Cytometric analysis. (**E**) Concentration of IL-12. Cytometric analysis. (**F**) Concentration of MCP-1. Cytometric analysis. (**G**) Concentration of IL-6. Cytometric analysis. Each bar represents mean ± SE (*N* = 3–8; * *p* < 0.05, ** *p* < 0.01, *** *p* < 0.001 vs. HO-1^+/+^ mice, # *p* < 0.05, ♂ vs. ♀).

Tumor growth and concentrations of cytokines in sera did not correlate. Although males grew much bigger tumors in lungs than females, concentrations of inflammatory and angiogenic factors were comparable in the sera of both sexes (Figure 5). Nevertheless, wild-type animals showed significantly lower concentrations of some cytokines than HO-1^+/−^ and HO-1^−/−^ mice. This effect was particularly visible in the case of IFNγ (Figure 5A) and KC (Figure 5B), both in males and females (*p* < 0.01 for IFNγ in males and *p* < 0.05 for IFNγ in females, *p* < 0.001 for KC in males and females). VEGF concentration was the highest in HO-1-deficient mice (*p* < 0.01 in males and *p* < 0.001 in females) (Figure 5C). A similar situation was seen in the case of IL-10 (Figure 5D) and IL-12 (Figure 5E), but statistical significance was reached only in males (*p* < 0.001 for IL-10 in males and *p* < 0.05 for IL-10 in females, *p* < 0.05 for IL-12 in males and *p* > 0.5 in females). There was no significant difference in the concentration of MCP-1 (*p* = 0.088 in males and *p* > 0.5 in females) (Figure 5F) and IL-6 (*p* > 0.2 in males and *p* > 0.5 in females) (Figure 5G).

### 3.7. Melanoma Growth in Lungs Affects Hematological Parameters in Mice

To investigate how tumor development influences hematological parameters in mice of different HO-1 genotypes, blood samples were collected three weeks after intravenous inoculation with B16(F10)(EGFP-I-Luc) melanoma cells and WBC, PLT, RBC, MCV, HGB, MCH, MCHC and HCT were measured using an ABC Vet^®^ analyzer. Blood samples from intact mice were used as controls.

**Figure 6:**
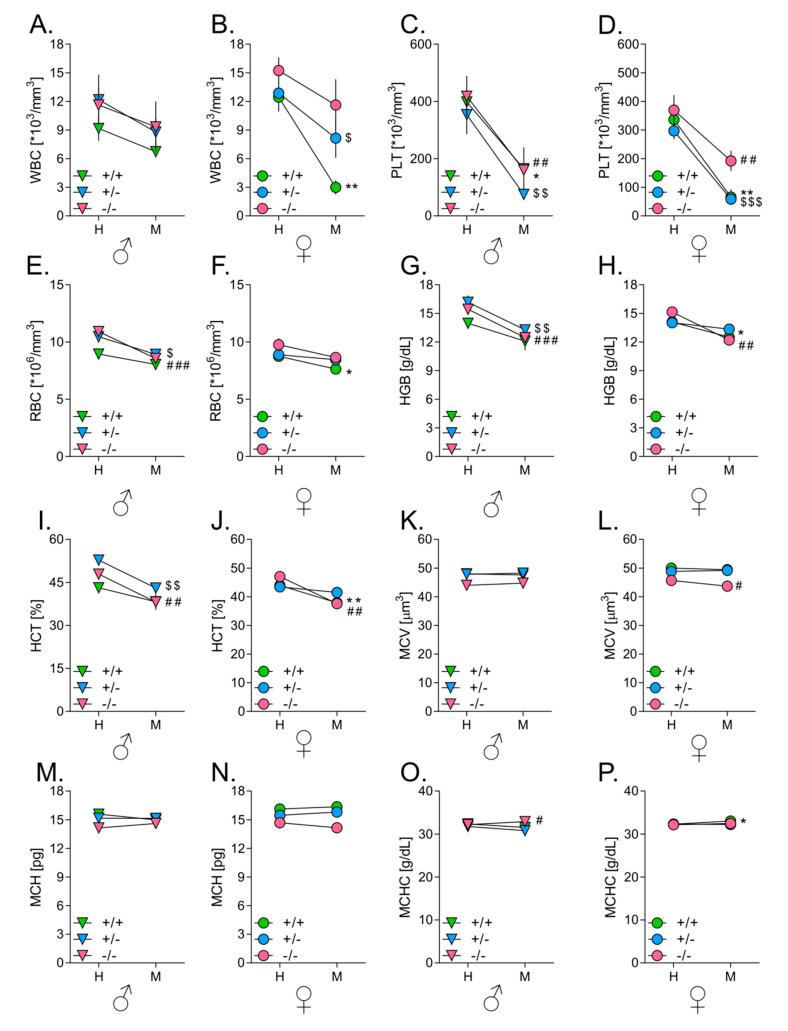
Effect of HO-1 expression in mice on hematological parameters. (**A**) White blood cells (WBC) in males. (**B**) WBC in females. (**C**) Platelets (PLT) in males. (**D**) PLT in females. (**E**) Red blood cells (RBC) in males. (**F**) RBC in females. (**G**) Hemoglobin (HGB) in males. (**H**) HGB in females. (**I**) Hematocrit (HCT) in males. (**J**) HCT in females. (**K**) Mean corpuscular volume (MCV) in males. (**L)** MCV in females. (**M**) Mean corpuscular volume (MCH) in males. (**N**) MCH in females. (**O**) Mean corpuscular hemoglobin concentration (MCHC) in males. (**P)** MCHC in females. Analysis of blood parameters by means of ABC Vet. Intact, Healthy (H) vs. Tumor-bearing, Metastatic (M). Each bar represents mean ± SE (*N* = 4–8; * *p* < 0.05, ** *p* < 0.01, tumor-bearing HO-1^+/+^ mice vs. intact HO-1^+/+^ mice; $ *p* < 0.05, $$ *p* < 0.01, $$$ *p* < 0.001 tumor-bearing HO-1^+/−^ mice vs. intact HO-1^+/−^ mice; # *p* < 0.05, ## *p* < 0.01, ### *p* < 0.001 tumor-bearing HO-1^−/−^ mice vs. intact HO-1^−/−^ mice).

Tumor development in all investigated animals led to decrease in circulating leukocyte numbers (Figure 6A,B). This decrease was the highest in HO-1^+/−^ (*p* < 0.05) and particularly visible in HO-1^+/+^ (*p* < 0.01) females, where values dropped below physiological standards. As shown by measurement with IVIS^®^ Lumina, these mice most effectively inhibited the growth of tumors in the lungs. In other groups, this tendency did not reach statistical significance (*p* = 0.135 in HO-1^−/−^ females, *p* = 0.071 in HO-1^+/+^ males, *p* = 0.084 in HO-1^+/−^ males and *p* = 0.256 in HO-1^−/−^ males).

In all mice, showing active homing of melanoma cells to lungs (and other organs), there was a significant reduction in numbers of platelets and erythrocytes in melanoma-bearing animals compared to healthy counterparts. The strongest effect was observed in animals that grew the biggest tumors—HO-1^+/−^ and HO-1^−/−^ males. Tumor growth was associated with lower levels of platelets (Figure 6C,D; females: *p* < 0.01 in HO-1^+/+^, *p* < 0.001 in HO-1^+/−^ and *p* < 0.01 in HO-1^−/−^; males: *p* < 0.05 in HO-1^+/+^, *p* < 0.01 in HO-1^+/−^ and *p* < 0.01 in HO-1^−/−^) and with anemia. Symptoms of anemia such as decreased numbers of erythrocytes (Figure 6E,F; females: *p* < 0.05 in HO-1^+/+^, *p* = 0.176 in HO-1^+/−^ and *p* = 0.073 in HO-1^−/−^; males: *p* = 0.084 in HO-1^+/+^, *p* < 0.01 in HO-1^+/−^ and *p* < 0.001 in HO-1^−/−^) decrease in concentrations of hemoglobin (*p* < 0.01 in HO-1^+/−^ and *p* < 0.001 in HO-1^−/−^) (Figure 6G,H), and values of hematocrit (*p* < 0.05 in HO-1^+/−^ and *p* < 0.01 in HO-1^−/−^) (Figure 6I,J) were largest in HO-1^+/−^ and HO-1^−/−^ males, the hosts that grew the biggest tumors. Finally, the size of erythrocytes (Figure 6K,L), amount of hemoglobin in erythrocytes (Figure 6M,N) and concentration of hemoglobin in erythrocytes (Figure 6O,P) were not affected by tumor development.

## 4. Discussion

Understanding the role of HO-1 in carcinogenesis is challenging due to the lack of distinction between its role in cancer cells and host cells. Previously, we showed that overexpression of HO-1 in murine B16(F10) melanoma cells increases their survival, proliferation and metastatic potential [15]. Here we investigated the role of HO-1 in host cells. We compared the growth of the B16(F10) cell line after inoculation to the hosts of different HO-1 genotypes: HO-1^+/+^, HO-1^+/−^ and HO-1^−/−^. Our results show that presence of HO-1 in host cells, including immune cells, can reduce growth and metastasis of melanoma. We found that: (1) females grew much smaller tumors than males after both intracutaneous and intravenous injection with melanoma cells, (2) growth of primary tumors and lung nodules was completely inhibited in HO-1^+/+^ females, which was associated with the augmented leukocyte infiltration of primary tumors with lymphocytes T as a major subpopulation, and (3) HO-1^+/^^−^ and HO-1^−/−^ males formed bigger primary tumors and more numerous lung nodules than their wild-type counterparts, as well as more of them showed micrometastases in liver and spleen.

Many conclusions regarding the effect of HO-1 on cell behavior are based on experiments using pharmacological activators (e.g., hem, CoPPIX, CoCl_2_) or inhibitors (e.g., SnPPIX, ZnPPIX, ZnMPIX, ZnDPIX). It is worth mentioning, however, that all these compounds show strong activities, that are unrelated to the activity of HO-1 [25]. Therefore, to avoid nonspecific effects of HO-1 modulators, we decided to use a genetic model, i.e., syngeneic mice with a different HO-1 genotype: HO-1^+/+^, HO-1^+/−^ and HO-1^−/−^.

Unexpectedly, we observed that tumors growing in females were evidently smaller than those in males both after intracutaneous and intravenous injection with melanoma cells, independently of the HO-1 genotype. Moreover, in many females, tumors disappeared after some time, suggesting successful activity of the immune system. To the best of our knowledge, similar observations have not been described previously and mechanisms underlining these observations are not yet known.

The most apparent reason for differences in tumor growth between males and females could be related to sex hormones. Coincidentally, the mortality caused by melanoma in humans is twice higher in males than in females [26,27]. The levels of estrogen also affect the final outcome of the disease, as post-menopausal women melanoma patients have poorer prognosis when compared to young, pre-menopausal women [28]. Interestingly, pregnancy and oral contraceptives are not considered factors influencing the incidence of cutaneous melanoma in women [29].

Growth of melanoma cells in vitro can be inhibited by sex steroid hormones: 17β-estradiol, progesterone, and dihydrotestosterone [30]. Furthermore, more aggressive melanomas exhibit lower levels of estrogen receptors: ER-α and ER-β mRNAs. ER-β expression is downregulated in tumor cells in comparison to surrounding healthy tissues in patients with diagnosed metastases in lymph nodes [31]. Additionally, the presence of ER-β in dysplastic lesions as well as in melanoma in situ may suggest the importance of the 17*β*-estradiol/ER-β pathway in the proliferation and transformation of melanocytes [32]. Moreover, B16(F10) cells display enhanced growth in ER-β knockout mice when compared to wild-type animals, what suggests the important role of estrogen signaling in protection against melanoma [33]. Interestingly, 17*β*-estradiol may induce dendritic morphology of the B16(F10) melanoma cell line [34]. It could play an important role in tumor growth and metastasis, as melanocytes’ transformation correlates with the loss of a dendritic shape and downregulation of cell surface markers [35]. The influence of estrogens on differentiation of melanoma cells may have one more important implication—it may favor the induction of immune response. Inflammatory infiltrations with lymphocytes and other cells, commonly occur in melanomas. Clinical analyses indicate that the more potent lymphocyte invasion is, the better is the prognosis for patients [36,37]. The immunogenicity of melanoma is related to the expression of MART-1, gp100, TRP-1, TRP-2 and tyrosinase, which affects the behavior of tumor-infiltrating lymphocytes, CD4^+^ T and CD8^+^ T. It has been demonstrated that the high expression of melanoma markers, especially gp100 epitopes, correlates with tumor regression and could improve immunotherapy [38]. In accordance, our results showed that tumors in females were more heavily infiltrated by leukocytes than in males. Moreover, the most dominant subpopulation of immune-infiltrating leukocyte in HO-1^+/+^ females was lymphocyte T. The proportion of these cells was also higher in females of other genotypes compared to corresponding males. The number of WBCs in the blood of females (again especially WT females) injecting with melanoma cells intravenously and growing lung tumors was much lower than in the blood of healthy mice. It may indicate for active mobilization of leukocytes from blood to tumor site. Finally, we observed that tumor tissues were darker in females than in males (data not shown), suggesting that they were more differentiated and therefore probably more immunogenic. Taking into consideration available data, we propose that sex hormones (mostly estrogens) could be responsible for the differences in tumor growth that we observe between female and male mice. However, this supposition needs to be further verified.

The fact that both wild-type males and females grew smaller tumors than HO-1-deficient animals may suggest the antitumor effect of HO-1 in host cells. Tumors from wild-type females were less heavily infiltrated with CD11b^+^Gr1^+^ myeloid cells. This population might include immature myeloid cells, as well as myeloid derived suppressor cells (MDSC), which can alter T cell response facilitating tumor growth [39,40]. Higher infiltration of T cells in the wild-type females than in the HO-1-deficient counterparts might also indicate that presence of HO-1 is required for the effective antitumor reaction of immune cells. In our previous study, we showed that carcinogen-induced lesions appeared earlier in HO-1-deficient mice than in wild-type animals, suggesting that HO-1 may protect healthy tissues against carcinogenesis [24]. Additionally, it has been shown that HO-1^−/−^ mice have higher numbers of CD4^+^ T cells and augmented production of Th-1 related cytokines: IL-1, IL-6, TNF-α and IFN-γ [41,42]. Therefore, the facilitated tumor growth in HO-1-deficient hosts might result from enhanced pro-inflammatory properties of tumor microenvironment. Indeed, the levels of IFN-γ, IL-6, KC, VEGF and MCP-1 in the sera of mice injected with melanoma cells both intracutaneously and intravenously were significantly higher in HO-1-deficient than in wild-type mice. Interestingly, inflammatory cytokines including IL-6, IL-10, IL-12p70, IFN-γ, TNF-α, MCP-1 and KC were previously shown to be differentially expressed in male and female C57 BL/6J mice in the melanoma model [43].

IFN-γ is an important activator of macrophages and is mostly known for its anti-viral activity. Interestingly, in the context of melanoma, loss of IFN-γ was shown to play a role in resistance toward immunotherapies [44]. The role of HO-1 in cancer recognition by NK cells is still poorly investigated. However, recent research has demonstrated that blocking HO-1 in various cervical cancer cell lines augmented the expression of INF-γ and TNF-α in the co-cultured NK cells and restored the expression of NK cell markers, namely: NKG2D, NKp30 and NKp46 [45]. In our studies, HO-1-deficient mice had elevated levels of IFN-γ, regardless of the sex; thus, the HO-1 status was the predominant factor regulating IFN-γ levels.

IL-12 mediates tumor suppression in many murine tumor models, including melanoma [46,47]. Induction of HO-1 was shown to decrease IL-12p70 levels in dendritic cells [48]. We observed similar phenomenon in the plasma of WT animals injected intravenously with melanoma cells, where the levels of IL-12p70 were inversely correlated with the levels of HO-1 in the stroma. However, the concentrations of IL-12 were not correlated with the inhibition of tumors; thus, IL-12 is not the mediator of tumor growth in our model.

Expression of IL-6 is associated with increased HO-1 expression in multiple myeloma (MM) and high levels of both (IL-6 and HO-1) can be used as a marker of poor prognosis in MM [49]. Moreover, in melanoma patients, high levels of IL-6 in the sera are associated with poor prognosis [50,51]. In our study, the level of IL-6 was not associated with the survival of mice, but we found that IL-6 levels were significantly higher in females than in males. Interestingly, a recent report showed that the long-term survival of melanoma patients immunized with a Hyper-IL-6-modified allogeneic whole-cell vaccine was improved [52]. It may suggest that IL-6 may enhance immunogenicity of melanoma cells.

HO-1 is constitutively expressed in T_reg_ (CD4^+^CD25^+^) cells [53] and the expression of HO-1 in antigen-presenting cells is necessary for proper actions of T_reg_ [54]. Melanoma cells can inhibit T cell proliferation through IL-10 and IFN-γ induction, in a T_reg_-dependent manner [55,56]. The overexpression of IL-10 in B16(F10) cells accelerates tumor growth through reduced presentation of melanoma antigens [57]. Indeed, many studies suggest that the presence of T_reg_ correlates with tumor progression. T_reg_ cells can recognize specific melanoma markers gp100 and TRP-1, produce IL-10 and inhibit anti-tumor response of T lymphocytes CD4^+^CD25^−^ [58]. Furthermore, recent studies indicate that HO-1 plays a role in polarizing macrophages towards the M2 phenotype, which is pro-angiogenic and produces IL-10. Macrophages that express HO-1 suppress the immune response and are considered a poor prognostic indicator in cancer patients (reviewed in [59]). In our experiments, the levels of IFN-γ and IL-10 were the highest in HO-1^−/−^ mice, which suggests that these cytokines may facilitate tumor growth.

Using the intravenous model of inoculation, we demonstrated that low levels or lack of HO-1 in host cells facilitates melanoma homing to different organs. The presence of GFP and luciferase reporter proteins in B16(F10)(EGFP-I-Luc) cells enabled us not only to detect micrometastases in tissue homogenates (by means of measurement of luciferase activity), but also to monitor tumor growth in living animals (using IVIS system). More effective response against cells injected intravenously could result from the use of B16(F10)(EGFP-I-Luc) cells that had more differentiated phenotype than wild-type cells (data not shown), used for intracutaneous inoculation. GFP can possess immunogenic properties and act as an adjuvant in activation of cytotoxic T lymphocytes against melanoma [60]. In agreement with our previous study [15], melanoma cells injected intravenously colonized mainly lungs, although we were able to detect them also in the liver, spleen, intestine, kidneys, and circulating in the blood. HO-1 deficiency predisposed to the formation of micrometastases in the livers, lungs and spleens. In accordance with this, it was recently demonstrated that HO-1 overexpression in normal tissues could inhibit lung melanoma metastases and it was correlated with decreased numbers of WBCs [61]. It is worth noting, that no metastases in HO-1^−/−^ recipients was observed in the kidneys, which is likely due to renal injury caused by iron accumulations typical for HO-1^−/−^ mice [62].

In our study, the increased micrometastases in HO-1-deficient mice might be connected with increased levels of VEGF and MCP-1. We previously discovered that HO-1 upregulates production of VEGF in endothelial cells [63] and that overexpression of HO-1 in melanoma cells increases the levels of VEGF leading to increased angiogenesis in tumors [15]. Melanoma cells are an important source of VEGF; thus, the observed low concentrations of VEGF in the blood of females developing tumors after intracutaneous administration of B16(F10) cells could result from reduced tumor growth. After the intravenous administration of melanoma cells, the levels of VEGF were elevated in HO-1^−/−^ animals, which was accompanied by a tendency toward higher number of micrometastases in HO-1-deficient animals (in males). This indicates that HO-1 deficiency might act in favor of tumor invasion, which is reflected in the higher levels of VEGF in the serum.

It is known that HO-1^−/−^-deficient animals have elevated levels of MCP-1 in their plasma [64]. Interestingly, deficiency of MCP-1 is associated with enhanced melanoma growth and an increase in lung metastases [65]. Coherently, our data show that HO-1^−/−^ animals (males) display increased MCP-1 levels, which was associated with increased lung metastases after intravenous administration of B16(F10) cells. Thus, MCP-1 could be one of the mediators of increased lung metastases in HO-1^−/−^ animals.

## 5. Conclusions

In summary, our results show that the presence of HO-1 in host cells, including tumor-infiltrating immune cells, can reduce the growth and metastasis of melanoma. Inhibition of tumor growth can be associated with stronger infiltration of tumors by T cells. Therefore, levels of HO-1 expression in host cells could be considered a prognostic factor, whereas, in growing tumors, they could be considered a target of anticancer therapy. Additionally, we observed the inhibition of tumor growth in females, supporting the notion that sex is an important factor affecting melanoma development.

## Supplementary Materials

The following are available online at https://www.mdpi.com/2076-3921/9/12/1223/s1, Figure S1. Effect of HO-1 expression in mice on primary tumor growth. Tumor volume in males (m) (*N* = 4–10) (**A**) and females (f) (*N* = 4–10) (**B**) of different HO-1 genotype: +/+, +/− or −/−. Each point represents mean ± SE.
