# Supplementary material for: Effect of Heme Oxygenase-1 on Melanoma Development in Mice—Role of Tumor-Infiltrating Immune Cells
Source: Antioxidants (Basel). 2020 Dec 3;9(12):1223. doi: 10.3390/antiox9121223 (PMC7761646; doi:10.3390/antiox9121223)
Supplement: Supplementary file 1 [file antioxidants-09-01223-s001.pdf]

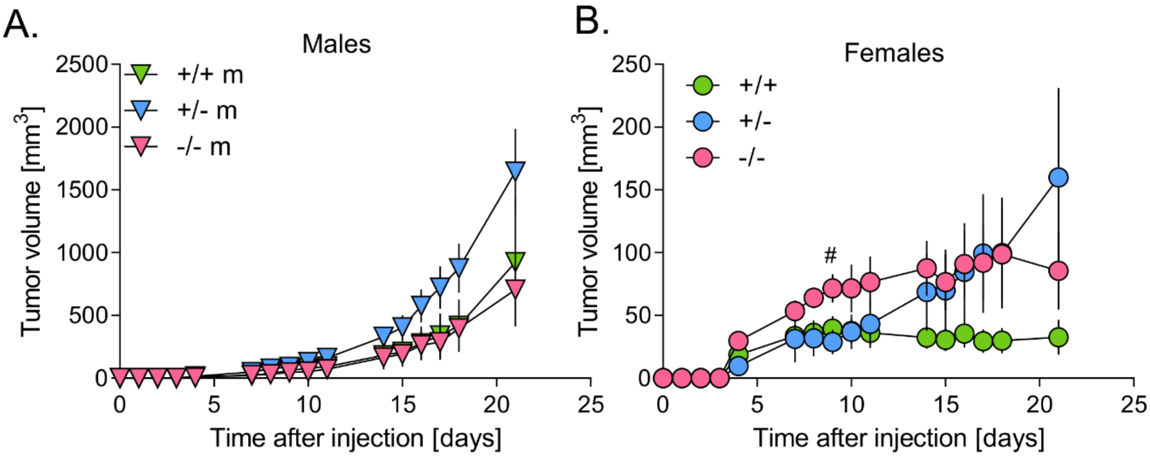

Figure S1. Effect of HO-1 expression in mice on primary tumor growth. Tumor volume in males (m) (*N* = 4–10) (**A**) and females (f) (*N* = 4–10) (**B**) of different HO-1 genotype: +/+, +/− or −/−. Each point represents mean ± SE.
